# Supplementary material for: ZIF-8-Derived Hollow Carbon for Efficient Adsorption of Antibiotics
Source: Nanomaterials (Basel). 2019 Jan 18;9(1):117. doi: 10.3390/nano9010117 (PMC6358953; doi:10.3390/nano9010117)
Supplement: Supplementary file 1 [file nanomaterials-09-00117-s001.pdf]

Support Information

ZIF-8-Derived Hollow Carbon for Efficient Adsorption of Antibiotics

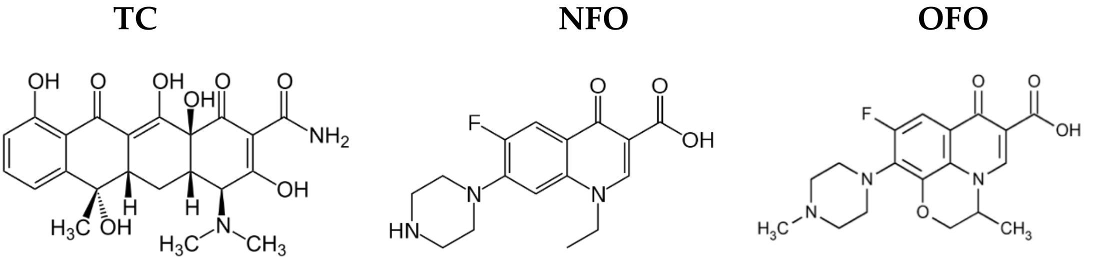

Figure S1. The chemical structure of TC, NFO, and OFO.

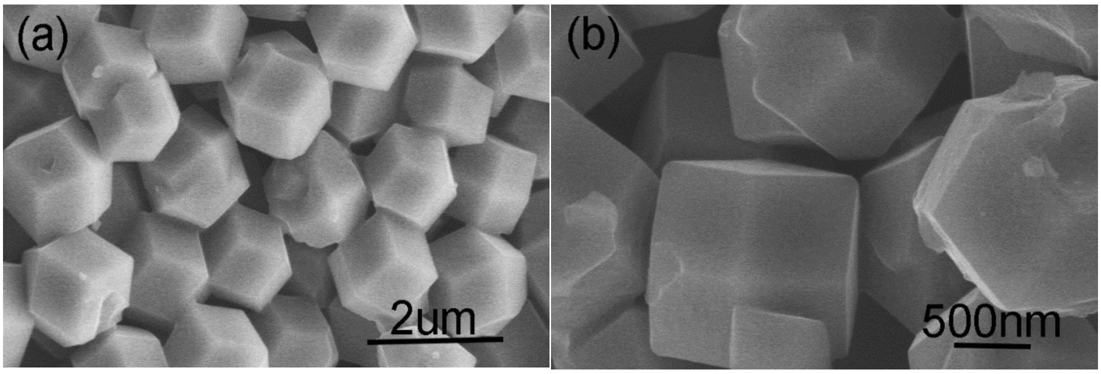

Figure S2. The Low-(**a**) and High-magnification (**b**) SEM images of the synthesized ZIF-8 without CTAB.

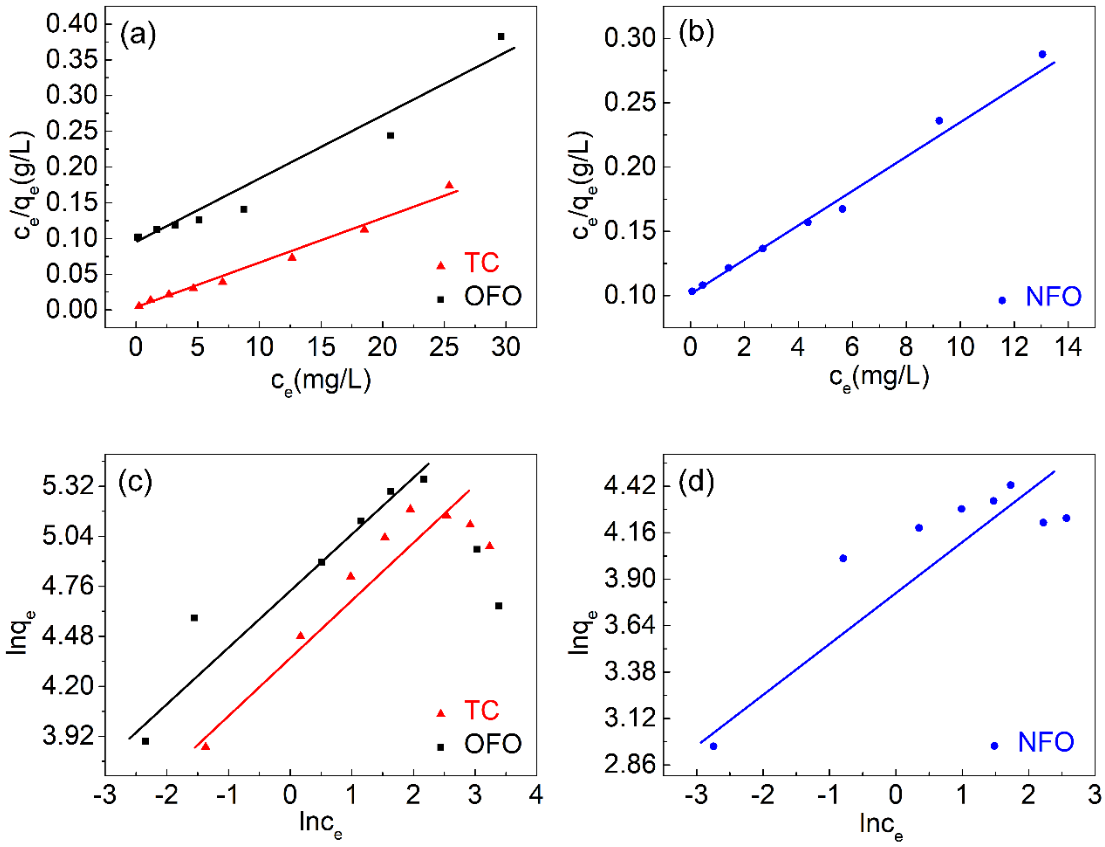

Figure S3. The isothermal adsorption fitting curves of Langmuir (**a**,**b**) and Freundlich (**c**,**d**) for TC, OFO and NFO by ZIF-8.

Figure S3 describe the isothermal adsorption fitting curves of Langmuir (Fig S3a, 3b) and Freundlich (Fig S3c, S3d) for TC, OFO and NFO by pure ZIF-8. Langmuir calculation showed that the maximum adsorption capacity of pure ZIF-8 for TC, OFO and NFO was 119.04, 111.48 and 38.69 mg g^{-1}, respectively.
